# Prediction of neoadjuvant therapy response in breast cancer based on interpretable artificial intelligence

**DOI:** 10.1097/JS9.0000000000003326

**Published:** 2025-09-24

**Authors:** Yao Zhou, Xin Shu, Fan Wang, Hui Xu, Hong-Qun Tang, Hao Fang, Jing Huang, Yi-Wei Wang, Hong-Liang Ji, Shi-Wei Zhang, Wei Qu, Jian-Hong Tu, Fan Niu, Li-Bin Deng

**Affiliations:** a Data Governance Center, Nanchang People’s Hospital, Nanchang, China; b School of Public Health, Jiangxi Medical College, Nanchang University, Nanchang, China; c Jiangxi Provincial Key Laboratory of Disease Prevention and Public Health, Nanchang University, Nanchang, China; d Yizhichu Medical Pathology Diagnosis Management Co., Ltd, Nanchang, China; e Affiliated Rehabilitation Hospital, Jiangxi Medical College, Nanchang University, Nanchang, China; f Department of Pathology, Hubei Provincial Hospital of Integrated Chinese and Western Medicine, Wuhan, China; g Pathology Department, The Nanchang People’s Hospital, Nanchang, China

**Keywords:** breast cancer, evaluation of therapeutic effect, explainable artificial intelligence, neoadjuvant therapy, tumor microenvironment

## Abstract

**Background:**

To develop an AI-based predictive model for neoadjuvant therapy (NAT) efficacy in breast cancer, we integrated multimodal data and analyzed tumor microenvironment (TME) features to provide interpretability.

**Methods:**

We retrospectively analyzed H&E-stained whole-slide images (WSIs) from a multicenter cohort of breast cancer patients receiving NAT to develop an AI predictive model. The cohort was stratified into training, test, internal validation, and external validation sets. Feature extraction used UNI and classification employed a multiple instance learning (MIL) framework. Model performance was evaluated via ROC curve analysis (AUC, precision, specificity, recall). Molecular mechanisms underlying model predictions were explored using TCGA multimodal data, integrating differential gene expression profiling with pathway enrichment analysis (GO, KEGG). TME component correlations with model scores were also investigated.

**Results:**

The AI model demonstrated robust discriminative capacity across three residual cancer burden (RCB)-based classification tasks in 826 patients from two centers, achieving peak performance in subtask 2 (NAT-sensitive: RCB 0–1 vs. NAT-resistant: RCB 2–3). For subtask 2, AUCs were 0.901 (training), 0.858 (test), 0.808 (internal validation), and 0.819 (external validation). Molecular analysis linked the model’s predictive efficacy to tumor cell cycle processes. TME analysis revealed positive correlations between model scores and activated immune cells (M0/M1 macrophages, dendritic cells), and negative correlations with inhibitory cells (M2 macrophages, resting mast cells). Crucially, the model’s predictive scores were closely related to tumor-infiltrating lymphocytes (TILs), with spatial colocalization observed between classification weights and TILs distribution. Significant differences in TILs levels occurred across model score strata, validating the model’s biological plausibility in predicting NAT response mechanisms.

**Conclusion:**

We developed an interpretable AI model that predicts response to neoadjuvant therapy in breast cancer using H&E slides. The model’s predictions are biologically interpretable, correlating with TME dynamics and spatial TIL patterns, offering a novel strategy for personalizing NAT treatment strategies.

**HIGHLIGHTS**

- Development of an artificial intelligence-based predictive model for neoadjuvant therapy response in breast cancer using multicenter pre-chemotherapy pathological histopathology images.
- Integrating multimodal data to elucidate the mechanisms underlying the model predictions of therapeutic efficacy.
- Providing the biological interpretability of the tumor microenvironment.

## Introduction

Breast cancer (BC) is one of the most common cancers among women globally, with persistently high incidence and mortality rates^[1,2]^. The mortality rate of BC is primarily attributed to patients with locally advanced and metastatic disease^[2,3]^. At present, neoadjuvant therapy (NAT) has become the standard treatment strategy for patients with locally advanced breast cancer^[4]^. By reducing tumor volume and downstaging the disease preoperatively, NAT aims to increase the rate of surgical resection, enhance the possibility of breast-conserving surgery, and decrease the risk of postoperative recurrence^[5,6]^. For patients with locally advanced disease who have been confirmed as malignant with metastasis through biopsy, direct surgery often fails to achieve satisfactory therapeutic outcomes and may even promote further dissemination of tumor cells^[6,7]^.

Nevertheless, current clinical practice faces a key bottleneck: breast cancer is remarkably heterogeneous, and its response to NAT is highly variable among patients^[4]^. Pathological examination is currently the mainstay for evaluating the efficacy of NAT, with the residual cancer burden (RCB) score used for quantitative assessment^[8,9]^. Only 19–60% achieve pathologic complete response (pCR), and 5–20% experience disease progression^[8-10]^. Therefore, there is an urgent clinical need for a scheme that can accurately predict the efficacy of NAT before breast cancer surgery to better guide the extent of surgical resection^[10]^. Moreover, although postoperative pathological assessment using the RCB score can accurately quantify residual tumor burden, its retrospective nature prevents surgeons from precisely planning the surgical extent preoperatively^[6]^. Statistics show that approximately 35% of patients require reoperation due to insufficient surgical margins, while 24% of patients suffer from complications related to overtreatment^[11,12]^.

Given these challenges, accurately predicting whether a patient will achieve pCR before undergoing NAT is crucial for decision-making in the treatment of locally advanced breast cancer^[13]^. This not only helps identify the patient population most likely to benefit from NAT but also avoids delaying more effective treatment options for patients due to inappropriate therapeutic choices^[11,13]^. The recent advancements in artificial intelligence (AI) technology have revolutionized the approaches to breast cancer management, demonstrating transformative potential across diagnostic precision, therapeutic intervention optimization, and prognostic assessment accuracy^[14,15]^. AI-based models, especially those integrating deep learning and multimodal data, have shown potential in improving the precision of predicting NAT response in breast cancer^[16–18]^. However, current AI models still face challenges in clinical application, such as the need to enhance model interpretability and generalizability^[16,19]^.

However, as the RCB score is determined postoperatively, it cannot guide the extent of surgical resection preoperatively^[3,20]^. With the increasing attention to the application of AI in the medical field, particularly in breast cancer treatment decision-making, there remains some controversy regarding whether to expand the scope of lymph node dissection intraoperatively^[21,22]^. Previous studies have proposed using pathological features combined with the RCB score to assess the efficacy of NAT in breast cancer, categorizing RCB 0–I as the group with significant treatment efficacy (NAT-sensitive) and RCB II–III as the group with less significant treatment response (NAT-resistant)^[20,22–24]^.

To address these challenges, this study innovatively employs pre-NAT biopsy samples for pathological grading prediction, establishing an intelligent predictive model that integrates multimodal data to correlate histopathological features with molecular mechanisms and tumor microenvironment (TME) dynamics. This methodology provides robust clinical decision support for personalized surgical planning prior to NAT in breast cancer management. Although clustering-constrained attention multiple instance learning (CLAM) has become the mainstream method for WSI analysis, its reliance on ImageNet-pretrained CNNs for patch feature extraction results in domain discrepancies between learned visual representations and histopathological morphology, ultimately constraining the model’s performance ceiling^[19,25]^. This study advances CLAM’s MIL framework and attention-based region screening mechanism by introducing the pathology foundation model UNI to replace conventional ImageNet CNNs^[19,26]^. Leveraging UNI’s generalizability from large-scale histopathology pretraining, we extract discriminative features that more faithfully capture tissue morphological patterns^[26]^. Furthermore, our model not only overcomes the temporal limitations inherent in conventional retrospective RCB assessment but also provides prospective predictions during neoadjuvant therapy planning, thereby offering valuable support for advancing precision treatment strategies.

## Materials and methods

This study adhered to the STROCSS (Strengthening the Reporting of Cohort, Cross-sectional and Case-control Studies in Surgery) criteria^[27,28]^ and TITAN (Transparency In The reporting of Artificial Intelligence) guidelines^[28,29]^.

### Sample acquisition

**Figure 1.**
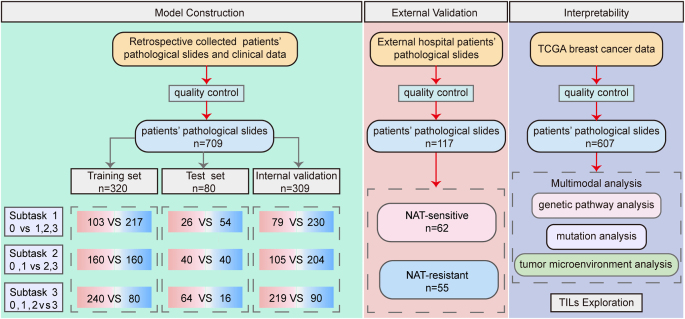
The data collection process of a pathomics-based AI model for predicting the prognosis of neoadjuvant therapy in breast cancer using WSI.

This study aims to establish a comprehensive, multicenter breast cancer specimen biobank to support multimodal pathological data analysis and intelligent predictive model development. We systematically collected extensive clinical and datasets on NAT across multiple dimensions, ensuring robust integration of histopathological, genomic, and therapeutic response profiles for advancing precision oncology strategies (Fig. 1).

### Hospital patients data collect

We retrospectively collected histopathological images and clinical data from 709 breast cancer patients who underwent NAT at the Nanchang People’s hospital (China). The dataset included gender, age, tumor location, Residual Cancer Burden (RCB) classification, Ki67 proliferation index, and expression levels of progesterone receptor (PR), estrogen receptor (ER), and androgen receptor (AR). These clinical parameters served as the primary data source for training and validating the intelligent predictive models (Table 1 and Supplementary Digital Content Table 1, available at: http://links.lww.com/JS9/F227).

**Table 1:** Baseline data of training set, validation set, and testing set

Characteristics	Training set	Testing set	Internal validation set
Number	320	80	309
Median age	50	51	50
Site			
Left breast	165 (51.56%)	36 (45.00%)	149 (48.22%)
Right breast	145 (45.31%)	43 (53.75%)	154 (49.84%)
Both breasts	10 (3.13%)	1 (1.25%)	6 (1.94%)
RCB			
0	103 (32.19%)	26 (32.5%)	79 (25.57%)
I	57 (17.81%)	14 (17.50%)	26 (8.41%)
II	80 (25.00%)	24 (30.00%)	114 (36.89%)
III	80 (25.00%)	16 (20.00%)	90 (29.13%)
Ki67			
≤20%	68 (21.25%)	19 (23.75%)	69 (22.33%)
>20%	252 (78.75%)	61 (76.25%)	240 (77.67%)
ER			
(−)	136 (42.50%)	32 (40.00%)	126 (40.78%)
(Weak)	24 (7.50%)	3 (3.75%)	19 (6.14%)
(Moderate)	21 (6.56%)	9 (11.25%)	24 (7.77%)
(Strong)	139 (43.44%)	36 (45.00%)	140 (45.31%)
PR			
(−)	184 (57.50%)	43 (53.75%)	169 (54.69%)
(Weak)	12 (3.75%)	1 (1.25%)	11 (3.56%)
(Moderate)	34 (10.62%)	10 (12.50%)	42 (13.59%)
(Strong)	90 (28.13%)	26 (32.50%)	87 (28.16%)
AR			
(−)	41 (12.81%)	12 (15.00%)	41 (13.27%)
(Weak)	24 (7.50%)	3 (3.75%)	17 (5.50%)
(Moderate)	69 (21.56%)	12 (15.00%)	71 (22.98%)
(Strong)	186 (58.13%)	53 (66.25%)	180 (58.25%)
HER2			
(−)	21 (6.56%)	8 (10.00%)	21 (6.80%)
(Weak)	52 (16.25%)	13 (16.25%)	56 (18.12%)
(Moderate)	111 (34.69%)	30 (37.50%)	126 (40.78%)
(Strong)	136 (42.50%)	29 (36.25%)	106 (34.30%)

Inclusion criteria: (1) Pathologically confirmed breast cancer; (2) Received NAT before surgery. Exclusion criteria: (1) Combined with other malignancies; (2) Special types of breast cancer; (3) Incomplete clinical or pathological data.

### TCGA multimodal data

We utilized a multimodal breast cancer (BRCA) dataset derived from The Cancer Genome Atlas, TCGA database (http://tcga.cancer.gov). Through rigorous quality control, we curated multidimensional data from 607 breast cancer patients, encompassing gene expression profiles, mutation spectra, tumor microenvironment composition, tumor-infiltrating lymphocytes quantification, and histopathological whole-slide images. This integrated resource comprehensively spans genomic patterns and morphological characteristics from histopathological imaging, enabling multi-omics correlation analysis for precision oncology research^[30]^.

### External validation sets data collect

We also collected histopathological images and clinical information data from 117 breast cancer patients undergoing NAT at Hubei Provincial Hospital of Integrated Traditional Chinese and Western Medicine as an external validation dataset. These external validation data samples further enriched our data sources, enhancing the diversity and representativeness of the cohort. Validation of the intelligent model across multi-hospital datasets allowed for a comprehensive evaluation of its generalizability, ensuring clinical applicability in diverse healthcare environments. Consecutive enrollment was applied to both the internal training, validation, and test datasets (all eligible patients during the study period) and the external validation dataset (from a contemporaneous, independent center), thereby minimizing selection bias.

### Pathological assessment

In accordance with the guidelines from the National Comprehensive Cancer Network (NCCN), patients received NAT and subsequently underwent surgery 2–3 weeks after the completion of NAT^[31]^. In pathological assessments, the status of the three hormone receptors was categorized as negative (−), weakly positive (weak), moderately positive (moderate), or strongly positive (strong)^[31]^. The residual cancer burden (RCB) scoring system was utilized to quantify residual disease by considering various factors, such as tumor bed size and the number of metastatic lymph nodes^[24]^. The RCB score was classified into four grades: (1) RCB 0, indicating no residual invasive cancer, equivalent to pathological complete response (pCR); (2) RCB I, representing minimal residual disease; (3) RCB II, indicating moderate residual disease; and (4) RCB III, denoting extensive residual disease^[6,24,32–34]^. And the immunohistochemical techniques were employed to evaluate four hormone receptors such as estrogen receptor (ER), progesterone receptor (PR), androgen receptor (AR), Human Epidermal growth factor Receptor 2 (HER2). The Ki-67 proliferation index was set at a cutoff value of 20%^[6,32,33]^.

### Image Quality Control and classification

To validate the quality and usability of pathological imaging data, we used OpenSlide-python 1.3.0 to segment the original whole slide images (WSI) of digital pathology slides. The segmentation size was set based on the actual physical distance: 64 μm × 64 μm. Specifically, at the maximum resolution layer of 20 ×, the images were segmented into patches of 256 pixels × 256 pixels. At the higher resolution layer of 40 ×, the images were segmented into patches of 512 pixels × 512 pixels. For enhanced data integrity, stringent quality control was applied to the segmented patches, excluding those failing size specifications (0-byte file size, image clarity score<185), overlapping multiple annotated regions, or containing pure background. These procedures ensured that only high-quality, standardized image patches entered downstream analytical workflows, thereby establishing a robust foundation for model accuracy and reliability.

### Model development

UNI is a general-purpose self-supervised AI model for pathology capable of learning robust visual features without the need for labeled data^[26]^. Its core advantage lies in its ability to automatically extract general features from large amounts of unlabeled image data, which is particularly important for processing complex pathological images. UNI can classify small regions within pathology images and identify regions of interest (ROIs), such as detecting the distribution of TILs in breast cancer. In this study, we introduced the UNI self-supervised learning AI model method based on DINOv2^[26]^. This method can efficiently extract valuable pathological features from a vast number of images without relying on manual annotation^[26]^. Based on this, we further applied the UNI AI model to extract features from pathology slides and then utilized the deep learning framework clustering-constrained attention multiple-instance learning (CLAM), which is specifically designed for pathology image analysis, to classify the extracted features^[19]^.

### Construction of efficacy prediction model

Based on the extracted pathological features and the RCB score used for efficacy assessment, we constructed a predictive model based on the deep learning framework CLAM, aiming to achieve precise prediction of the efficacy of NAT in breast cancer. The model adopted a data fusion architecture based on multiple instance learning (MIL), with the following workflow: image features were extracted from the patches using the optimized UNI; key features and heterogeneous regions related to treatment response were identified through a self-attention mechanism; and individual efficacy prediction scores were ultimately obtained within a machine learning framework. To ensure model generalizability, we implemented rigorous stratified 10-fold cross-validation throughout the study. Specifically, each fold was partitioned into training and test sets at an 8:2 ratio, guaranteeing sample-level independence and confirming the absence of significant overfitting. Through this rigorous training and testing process, we were able to comprehensively evaluate the model’s accuracy and generalizability, ensuring its reliability in real-world clinical applications.

### Model training process

Initially, tissue was delineated from the uniform background via saturation thresholding, retaining only pixels whose saturation exceeded the predefined threshold as tissue. Subsequently, non-overlapping 512 × 512-pixel tissue patches were harvested at the native 40 × magnification and down-sampled to 256 × 256 pixels at an effective 20 × magnification—a dimension we previously established as optimal for the UNI encoder in this setting^[26]^. Features were then extracted from these patches according to the specific protocol of each feature extractor, which customarily began with standard red–green–blue (RGB) color channels normalization^[35]^. Last, the resulting patch-level embeddings were forwarded to CLAM classifier trained independently for each feature extractor^[19]^. CLAM assigns trainable attention scores ranging from 0 to 1 to all patches within a slide, aggregates them into slide-level representations through attention-weighted averaging, and performs classification via a fully connected layer with one output node per class. Both training and inference adhered to the CLAM default pipeline; to enhance interpretability, the system concurrently generates an attention heat-map across the entire WSI, visually elucidating the model’s decision basis^[19]^. The data collection pipeline was complete; every enrolled WSI was accompanied by its paired H&E section and corresponding clinical label, with no instances of missing data. To mitigate cross-center variability, stringent per-channel RGB standardization was applied to all tissue patches prior to feature extraction. This eliminated scanner-specific bias between the UNIC PRECICE 610 × 1 and KFBio KF-PRO-005 WSI devices, yielding statistically indistinguishable feature distributions (*P* > 0.05) and maintaining identical downstream model performance, confirming negligible scanner-induced heterogeneity.

### Model evaluation

ROC Curve: The performance of the model under different thresholds was evaluated by plotting the ROC curve. The ROC curve uses the False Positive Rate (FPR) as the *x*-axis and the True Positive Rate (TPR) as the *y*-axis. AUC Value: The area under the curve (AUC) of the ROC was calculated. In addition, the stability and generalizability of the model were assessed through cross-validation and independent test sets.

### Tumor-infiltrating lymphocytes score

The evaluation of tumor-infiltrating lymphocytes (TILs) has gradually gained recognition for assessing the sensitivity of treatment outcomes in breast cancer patients receiving NAT^[31,36]^. Moreover, TILs are considered to have a positive prognostic role in patients with tumors that exhibit high biological invasiveness and are widely accepted as an indicator of immune activation^[36,37]^. Based on the clinical significance of TILs in breast cancer treatment, we conducted data analysis. In accordance with the recommendations of the International Immune Biomarker Working Group, TILs were assessed on hematoxylin and eosin (H&E)-stained slides of untreated primary tumors^[38]^. We also adopted the classification criteria for TILsl: low TILs (0–10%), intermediate TILs (11–59%), and high TILs (60–100%)^[37,39]^. TILs were subsequently analyzed as both continuous and categorical variables in our data analysis.

### Analysis of differentially expressed genes

Criteria for identifying significantly different genes were set at |log2(FC)| ≥ 1.0 and *P*-adjust < 0.05. Volcano plots and box-plot were generated utilizing the R package “ggplot2”.

### Ethical considerations

The related protocols for the retrospective study constituting the clinical platform of this research have been approved by the respective ethics committees of all centers. The data of the training set, test set, and internal validation set used for model construction have been approved by Nanchang People’s Hospital (Ethics Approval Number: K-kt2025026). The data ethics of the external validation set has been approved by Hubei Provincial Hospital of Integrated Traditional Chinese and Western Medicine (Ethics Approval Number: 2025 ethics review Document No. 028).

### Statistical analysis

In this study, the programming languages Python and R were utilized. For Python, version 3.9.12 was employed in combination with the Spyder development environment. The libraries used included OpenSlide, csv, pandas, Matplotlib, NumPy, OS, keras, and CV2, among others. For R, version 4.4.1 was used in conjunction with R-Studio, and the packages DESeq2 and limma were employed.

## Results

We retrospectively collected clinical data from 709 breast cancer patients treated with NAT, including gender, age, tumor laterality (left breast, right breast, bilateral), RCB classification (grades 0/I/II/III), Ki67 proliferation index, and expression status of progesterone receptor (PR), estrogen receptor (ER), androgen receptor (AR), and HER2. These clinical parameters formed the foundational dataset for developing the training, testing, and validation sets of the intelligent predictive model, as detailed in Table 1.

### Assessment of model performance.

To evaluate the predictive model, 709 breast cancer patients were divided into a training set (*n* = 320), a validation set (*n* = 80), and a test set (*n* = 309). The model was tested using three classification tasks based on four RCB score categories (0, 1, 2, and 3): subtask 1 (RCB 0 vs. RCB 1, 2, 3), subtask 2 (RCB 0 and 1 vs. RCB 2 and 3), and subtask 3 (RCB 0, 1, 2 vs. RCB 3) (Fig. 1).

**Figure 2.**
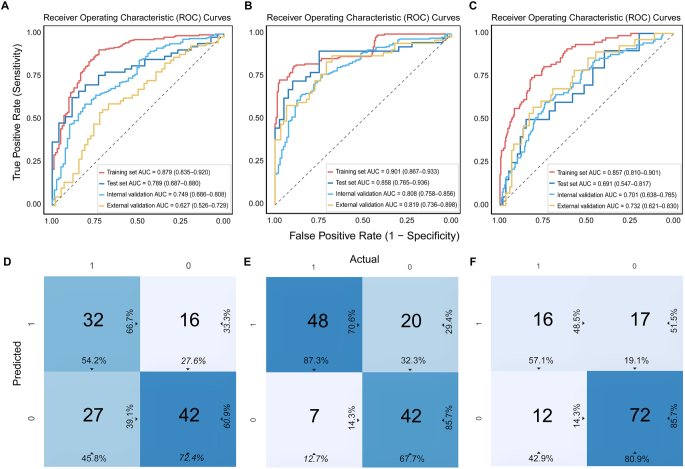
Receiver operating characteristic (ROC) curves for the predictive performance of three RCB classifications and the confusion matrix of the external validation dataset. (A) The ROC cures of subtask 1 (RCB 0 vs. RCB 1, 2, 3); (B) the ROC cures of subtask 2 (RCB 0 and 1 vs. RCB 2 and 3); (C) the ROC cures of subtask 3 (RCB 0, 1, 2 vs. RCB 3); (D) the confusion matrices of subtask 1; (E) the confusion matrices of subtask 2; (F) the confusion matrices of subtask 3.

As shown in Fig. 2A-C, the results indicated that subtask 1 (RCB 0 vs. RCB 1, 2, and 3) had a training set AUC of 0.879, a test set AUC of 0.789, an internal validation AUC of 0.749, and an external validation AUC of 0.627. Subtask 2 (RCB 0 and 1 vs. RCB 2 and 3) exhibited a training set AUC of 0.901, a test set AUC of 0.858, an internal validation AUC of 0.808, and an external validation AUC of 0.819. Subtask 3 (RCB 0, 1, and 2 vs. RCB 3) had a training set AUC of 0.857, a test set AUC of 0.691, an internal validation AUC of 0.701, and an external validation AUC of 0.732. The results suggested that subtask 2 (RCB 0 and 1 vs. RCB 2 and 3) demonstrated the best stability in model training and testing, outperforming subtask 1 and subtask 3.

To further evaluate the generalization performance of the models for three classification tasks using an external validation set, we analyzed their confusion matrices (Fig. 2D-F) and drew the following conclusions: In subtask 1, the model demonstrated moderate performance in distinguishing RCB 0 from other RCB categories, with a precision of 66.7%, recall of 54.2%, and specificity of 72.4%. These metrics indicate that while the model has a certain capability in correctly identifying samples of RCB 0, there is still room for improvement, particularly in reducing the number of false negatives (FN).

The model in subtask 2 exhibited the best performance in differentiating between RCB 0 and 1 and RCB 2 and 3, with a precision of 70.6%, a recall of 87.3%, and a specificity of 67.7%. This model’s high recall rate is notable, suggesting its ability to identify the majority of samples from RCB 0 and 1. These results indicate that the model in subtask 2 performs well in distinguishing between these two sets of RCB categories.

In contrast, the model in subtask 3, which was tasked with differentiating between RCB 0, 1, and 2 and RCB 3, showed relatively poor performance, with a precision of 48.5%, a recall of 57.1%, and a specificity of 80.9%. Despite the high specificity, the decrease in precision and recall suggests that the model faces challenges in accurately identifying RCB 3 samples, potentially due to significant differences in features between RCB 3 and other RCB categories, which may make it difficult for the model to make accurate distinctions. The analysis reveals that models across different groups exhibit varying levels of performance when distinguishing between different RCB categories, with the model in subtask 2 showing the best performance in terms of recall and precision when differentiating between RCB 0 and 1 and RCB 2 and 3.

Drawing on previous studies that used pathological features combined with RCB scores to assess the efficacy of NAT for breast cancer^[21,22,40]^, we defined RCB 0 and 1 as the group with significant treatment response (NAT-sensitive) and RCB 2 and 3 as the group with poor treatment response (NAT-resistant) in subtask 2^[20,23]^. Subsequent analyses were conducted based on this classification.

### Molecular mechanisms associated with the efficacy of neoadjuvant therapy

**Figure 3.**
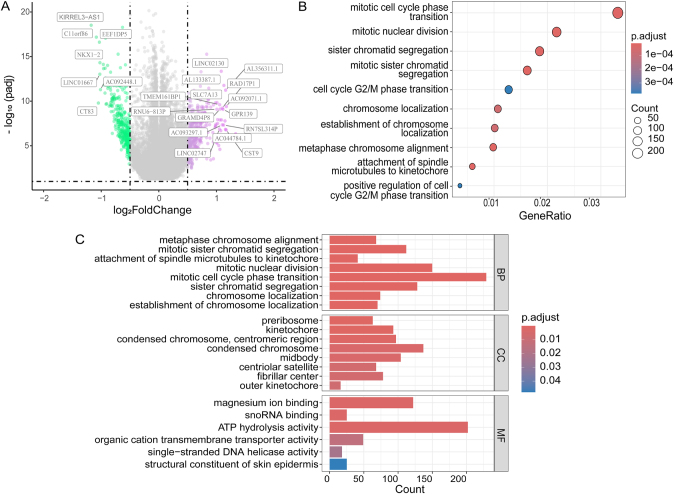
Differential gene expression analysis and KEGG/GO enrichment analysis related to NAT efficacy in breast cancer. (A) Volcano plot of differentially expressed genes; (B) KEGG pathway enrichment plot of differentially expressed genes; (C) GO enrichment analysis plot of differentially expressed genes.

By utilizing TCGA pathological images to categorize NAT-sensitive and NAT-resistant groups, and integrating TCGA breast cancer gene expression profiles, we investigated molecular pathways significantly associated with NAT response. As shown in Fig. 3A, differential analysis identified a set of differentially expressed genes (DEGs) linked to NAT efficacy in breast cancer. Notably, *KIRREL3-AS1, C11orf86*, and *EEF1DP5* were significantly downregulated. Existing studies indicate that *KIRREL3-AS1* and *C11orf86* act as prognostic markers for tumor staging and distant metastasis, underscoring their potential as novel therapeutic targets in breast cancer. The KEGG pathway enrichment analysis presented in this study (Fig. 3B) delineates the significantly enriched biological pathways associated with the identified differentially expressed genes.

Mitotic cell cycle phase transition and mitotic nuclear division pathways exhibit the highest generatio, indicating a substantial representation of differentially expressed genes within these processes. The significant enrichment in GO analysis (Fig. 3C) indicates that differentially expressed genes are enriched in the process of mitosis in cells, including key steps such as chromosome alignment, segregation, localization, and cell cycle regulation^[41]^. Abnormalities in these processes may lead to disordered cell division, thereby affecting the proliferation and genetic stability of breast cancer cells^[41,42]^. These findings indicate that these genes play a crucial role in the proliferation and genetic stability of breast cancer cells, potentially significantly influencing the improvement and optimization of NAT efficacy.

### Mutation types related to neoadjuvant therapy efficacy

**Figure 4.**
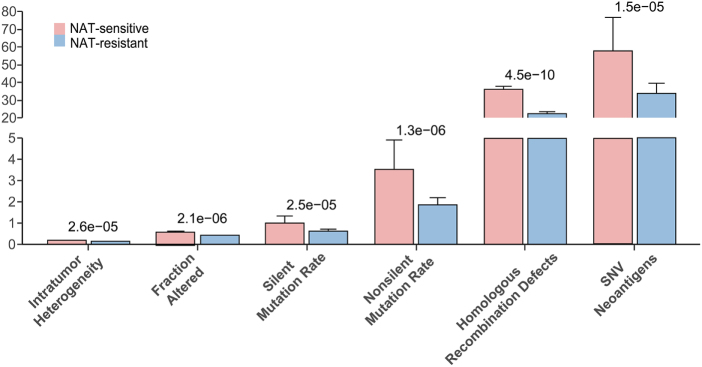
Utilizing a predictive model to analyze changes in molecular mechanism mutations in breast cancer during NAT.

Previous studies have demonstrated that specific mutation patterns are closely associated with the efficacy of NAT. Our analysis results revealed significant differences (*P* < 1 × 10^−4^) between the NAT-resistant and NAT-sensitive groups in intratumor heterogeneity, fraction altered, silent mutation rate, nonsilent mutation rate, homologous recombination deficiency (HRD), and single-nucleotide variation (SNV) neoantigens (Fig. 4). Notably, the most pronounced disparity was observed in HRD, with the NAT-sensitive group exhibiting a significantly higher HRD compared to the NAT-resistant group (*P* = 4.5 × 10^−10^). These findings suggest that alterations in homologous recombination deficiency (HRD) and related mutation types may effectively predict therapeutic response, further underscoring the importance of these mutations as potential biomarkers for personalized treatment strategies^[43]^.

### The composition of the tumor microenvironment associated with the efficacy of neoadjuvant therapy in breast cancer

The composition of the TME is considered a key factor influencing the efficacy of NAT in breast cancer^[41,42]^. We identified components significantly associated with molecular screening and treatment outcomes using data from TCGA^[30]^.

**Figure 5.**
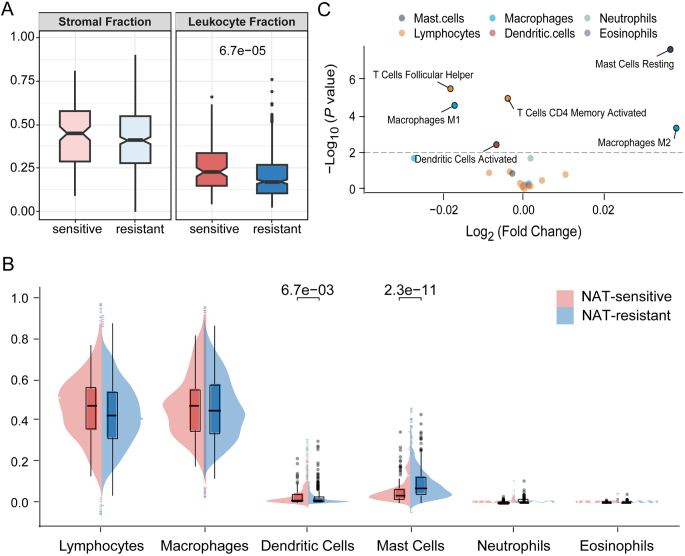
Screening of tumor microenvironment components associated with neoadjuvant therapy in breast cancer. (A) Expression profiles of stromal and leukocyte fractions in the tumor microenvironment of NAT-sensitive and NAT-resistant breast cancer samples; (B) expression levels of six immune cell types within the leukocyte compartment in NAT-sensitive and NAT-resistant breast cancer samples; (C) expression patterns of immune cell subsets in NAT-sensitive and NAT-resistant breast cancer samples.

In Fig. 5A, we illustrate the differences in the proportions of stromal fraction and leukocyte fraction within the tumor microenvironment of breast cancer samples classified as NAT-sensitive and NAT-resistant to NAT. The findings indicate that there is no significant difference in the stromal fraction between NAT-sensitive and NAT-resistant groups (*P* > 0.05). However, the leukocyte fraction is significantly higher in the NAT-sensitive group compared to the NAT-resistant group (*P* = 6.7 × 10^−5^).

We further examined the expression levels of six different types of immune cells within the lymphocytic component of NAT-sensitive and NAT-resistant breast cancer samples (Fig. 5B). As shown, the numbers of lymphocytes, macrophages, neutrophils, and eosinophils are slightly higher in the NAT-sensitive group compared to the NAT-resistant group, but these differences do not reach statistical significance (*P* > 0.05). In contrast, dendritic cells are significantly more abundant in the NAT-sensitive group (*P* = 6.7 × 10^−3^), while mast cells are significantly more highly expressed in the NAT-resistant group (*P* = 2.3 × 10^−11^). These findings suggest that the differential expression of specific immune cell subsets may be associated with the RCB response to NAT in breast cancer.

To further elucidate the interpretability of our model predictions, we conducted a differential analysis of immune cell subtypes (Fig. 5C). Our analysis revealed that, compared to the NAT-resistant group, the NAT-sensitive group exhibited higher expression of M1 macrophages, follicular helper T cells, activated CD4 memory T cells, and activated dendritic cells (*P* < 0.01). On the one hand, this indicates that the immune microenvironment in the NAT-sensitive group is activated, which is conducive to the prognosis of breast cancer patients when predicting RCB grades 0 and 1. On the other hand, the NAT-resistant group exhibits relatively higher expression of immunosuppressive M2 macrophages and resting mast cells compared to the NAT-sensitive group (*P* < 0.01); these cells are associated with immune suppression and may promote oncogenesis, suggesting that the immune microenvironment in the NAT-resistant group is suppressed, which is detrimental to patient prognosis^[44]^.

### Association of neoadjuvant therapy efficacy with TILs in breast cancer patients

**Figure 6.**
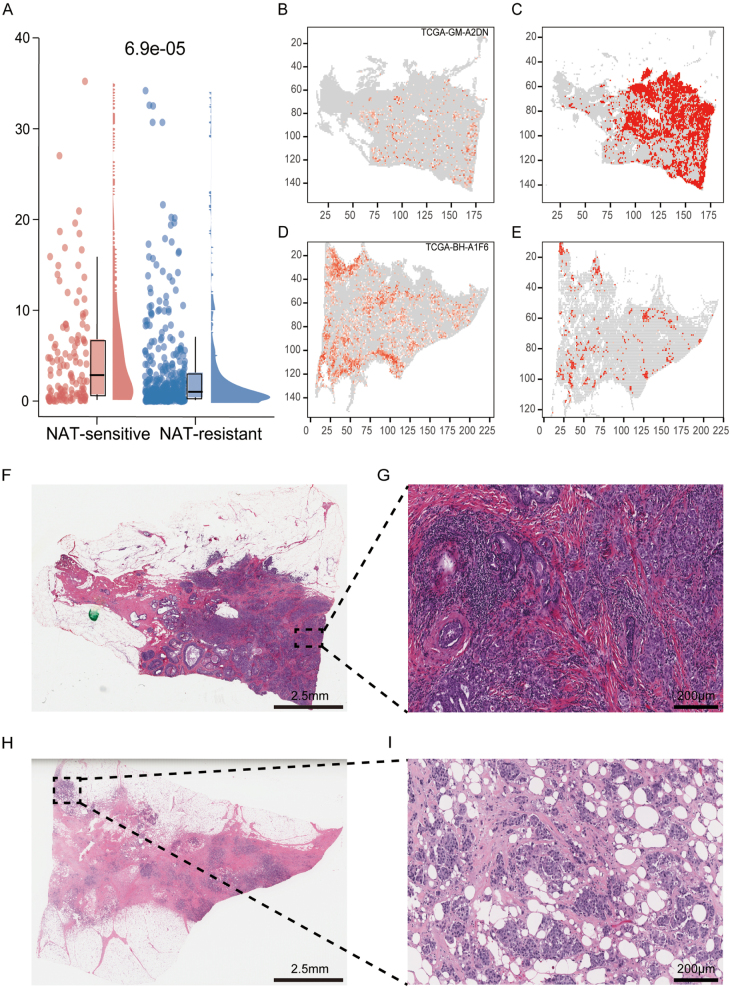
TILs score in breast cancer patients receiving NAT. (A) Comparison of TILs scores between NAT-sensitive and NAT-resistant groups; (B) classification weight map for NAT-sensitive group; (C) lymphocyte aggregation areas in NAT-sensitive group; (D) classification weight map for NAT-resistant group; (E) lymphocyte aggregation areas in NAT-resistant group; (F) low magnification H&E staining of NAT-sensitive group (magnification × 1); (G) high magnification H&E staining of NAT-sensitive group (magnification × 10); (H) low magnification H&E staining of NAT-resistant group (magnification × 1); (I) high magnification H&E staining of NAT-resistant group (magnification × 10).

We further explored the potential of our developed model in predicting the spatial distribution of TILs within the TME of breast cancer patients undergoing NAT and its correlation with patient prognosis. Through quantitative analysis, we compared TILs scores between NAT-sensitive and NAT-resistant groups, revealing a significant difference (*P* = 6.9 × 10^−5^), with notably higher TILs scores in the NAT-sensitive group (Fig. 6A). The results respectively present classification weight maps for assessing patient sensitivity to NAT, corresponding to the NAT-sensitive and NAT-resistant groups (Fig. 6B and 6D). We have depicted the spatial aggregation patterns of lymphocytes within the tumor microenvironment in the Fig. 6C and 6E. In the NAT-sensitive group with superior therapeutic outcomes, significant lymphocyte clustering is observed (Fig. 6C), while the NAT-resistant group with suboptimal therapeutic responses demonstrates markedly reduced lymphocyte aggregation (Fig. 6E). This spatial contrast visually corroborates the quantitative correlation between lymphocyte infiltration density and NAT efficacy. This suggests that a higher TILs score and greater lymphocyte aggregation are closely related to a better prognosis with NAT^[31,38]^. Supporting studies indicate that the degree of lymphocyte infiltration within tumors is typically positively correlated with the efficacy of immune checkpoint inhibitors, with higher infiltration levels associated with better therapeutic outcomes^[45,46]^. H&E staining results further confirm this: significant lymphocyte infiltration is observed at the tumor margins in the NAT-sensitive group (Fig. 6F and 6G), while the NAT-resistant group shows a marked reduction in lymphocyte infiltration (Fig. 6H and 6I). These findings suggest that the spatial aggregation of TILs may influence the efficacy of NAT by enhancing immune recognition efficiency^[31,45]^. Specifically, in the NAT-sensitive tumor microenvironment, active lymphocyte infiltration is associated with better patient prognosis, whereas in the resistant group, reduced lymphocyte infiltration is associated with poorer prognosis outcomes^[46]^

**Figure 7.**
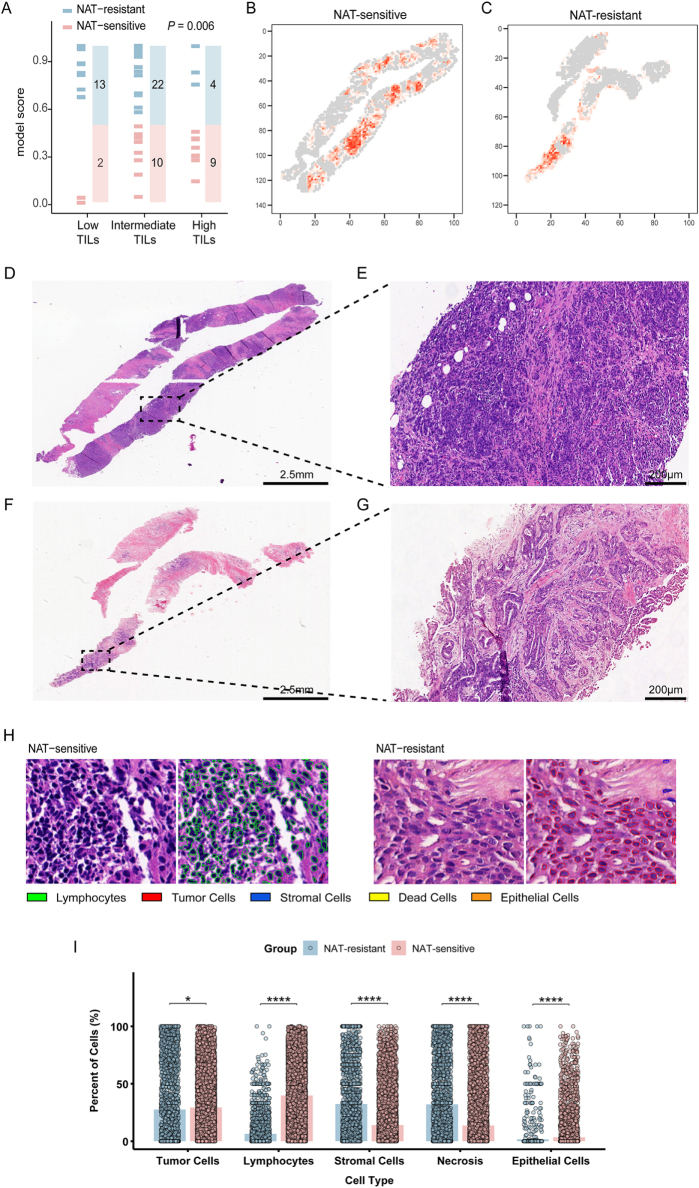
Results from the external validation cohort on the correlation between sensitivity to NAT and the distribution characteristics of TILs in breast cancer. (A) Analysis of breast cancer patient grouping based on TILs percentage and NAT sensitivity. TILs are categorized into three levels: low TILs percentage (0–10%), intermediate TILs percentage (11–59%), and high TILs percentage (60–100%); (B) lymphocyte distribution weight map of the tumor microenvironment in the NAT-sensitive group; (C) lymphocyte distribution weight map of the tumor microenvironment in the NAT-resistant group; (D) low-magnification H&E staining section of tumor tissue in the NAT-sensitive group (magnification × 1); (E) high-magnification H&E staining section of tumor tissue in the NAT-sensitive group, showing the distribution of lymphocyte aggregation (magnification × 10); (F) low-magnification H&E staining section of tumor tissue in the NAT-resistant group (magnification × 1); (G) high-magnification H&E staining section of tumor tissue in the NAT-resistant group, showing sparse distribution of lymphocytes (magnification × 10). (H) Exemplar high attention patches from NAT-sensitive group and NAT-resistant group cases with corresponding cell labels. (I) Quantitative cellular profiling of the NAT-sensitive and NAT-resistant groups within the highly attended regions revealed comparative changes in tumor, lymphocytes, stromal cells, and necrosis and epithelial cells.

Using an external validation dataset, we further systematically revealed the dynamic correlation between sensitivity to NAT and the distribution characteristics of TILs in breast cancer (Fig. 7A). This correlation exhibits a gradient difference among various response groups. Consistent with the previous studies, we revealed a significant correlation between TILs abundance and NAT sensitivity (Fig. 7A, *P* = 0.006) using a three-tier classification (low TILs: 0–10%; intermediate TILs: 11–59%; high TILs: 60–100%)^[37]^. Specifically, the proportion of High TILs in the NAT-sensitive group reached 69.2%, compared to only 30.7% in the resistant group (Fig. 7A). Further analysis indicated that NAT-sensitive group and NAT-resistant group used weight maps to quantify the differentiated spatial distribution patterns of TILs in the tumor core and stroma (Fig. 7B and 7C). The results indicated that the NAT-sensitive group showed an extensive “abundant-infiltration” pattern, while the resistant group exhibited a “sparse” feature, which may affect the interaction efficiency between immune cells and tumor cells^[37]^. Histologically, through H&E staining demonstrated the typical phenomenon in the NAT-sensitive group where tumor cell nests were surrounded by continuous dense lymphocytes (Fig. 7D and 7E); in contrast, the tumor–stromal junction in the NAT-resistant group only had a few scattered lymphocyte clusters (Fig. 7F and 7G). These findings further confirm the importance of TILs spatial distribution characteristics in predicting the efficacy of NAT. To elucidate the biological interpretability of the histopathological regions on which the model focuses, we performed cell-level semantic segmentation of the high attention regions within whole-slide images from the NAT-sensitive and NAT-resistant groups. As shown in Fig. 7H, attention-weighted interpretability maps intuitively reveal dense immune-cell infiltrates (green) in the NAT-sensitive group, whereas the model predominantly highlights tumor-cell-enriched regions (red) in the NAT-resistant group. Subsequently, we quantitatively characterized the cellular composition of these high-attention regions (Fig. 7I). Lymphocyte and epithelial cell proportions were markedly higher in the NAT-sensitive group than in the NAT-resistant group (*P* = 0 and *P* = 3.73 × 10^−12^, respectively), indicating that an active immune microenvironment is closely associated with favorable NAT response and improved patient prognosis. Conversely, the proportion of tumor cells within the high attention regions was paradoxically lower in the NAT-resistant group (*P* = 2.43 × 10^−2^), likely because abundant stromal and necrotic components dilute the effective tumor content. Further analyses demonstrated significantly elevated proportions of stromal cells and necrotic areas in the NAT-resistant group (*P* = 7.55 × 10^−111^ and *P* = 3.02 × 10^−104^, respectively), implicating fibrosis, immune exclusion, or hypoxic microenvironments in attenuating NAT efficacy. Collectively, the model precisely captures key histological determinants of therapeutic response through its high attention weights, providing a reproducible and interpretable quantitative framework for dissecting the mechanisms underlying NAT response at the cellular level.

## Discussion

The traditional RCB score is a key indicator for evaluating the efficacy of NAT in breast cancer^[8]^. However, its retrospective nature based on postoperative pathological assessment limits its preoperative application value^[4,23]^. This study integrates multimodal data to develop an AI-based predictive model for preoperative precise evaluation of breast cancer response to NAT, optimizing surgical planning and providing a reference for clinicians to select NAT drugs^[26,44,47]^.

This study integrates multimodal data from prechemotherapy biopsy samples to construct an AI-based RCB grading prediction model for breast cancer NAT^[13,48,49]^. The model performed differently in three RCB classification tasks^[8]^: subtask 1 (RCB 0 vs. RCB 1-3), subtask 2 (RCB 0 and 1 vs. RCB 2 and 3), and subtask 3 (RCB 0-2 vs. RCB 3). Among them, subtask 2 performed the best, with an AUC value of 0.901 in the training set and 0.819 in the external validation set, demonstrating good generalization ability. This result is in line with the classification strategy in previous studies, which limited conservative surgery to RCB 0 and 1 levels and guided expanded surgical treatment for breast cancer at RCB 2 and 3 levels^[6,8,33]^. This precise preoperative predictive ability provides important decision-making support for surgeons, enabling them to develop personalized surgical plans before the start of NAT^[6,11,50,51]^. For patients predicted to achieve pathological complete response (pCR, RCB 0/1 level), they can be considered for entry into observational clinical trials to avoid unnecessary surgical trauma^[6,7]^. For high-risk RCB 2/3 patients, positive lymph node regions can be marked preoperatively to precisely guide the scope of axillary lymph node dissection or to adjust treatment methods in a timely manner, thereby improving treatment outcomes and patient prognosis^[3,7,23]^.

The TME, a complex ecosystem comprising a variety of cell types including immune cells, fibroblasts, and endothelial cells as well as extracellular matrix components^[42]^, plays a key role in determining the response to NAT^[41]^. The microenvironment prediction model constructed in this study can accurately assess the tumor microenvironment characteristics of breast cancer patients^[52]^. By analyzing indicators such as tumor immune cell infiltration and immune cell subtypes in the tumor microenvironment^[30]^, we found that patients with different RCB grades have different microenvironmental characteristics. Patients in the NAT-sensitive group (RCB 0/1 level) often have higher levels of TILs and more active immune responses, while the microenvironment of patients in the NAT-resistant group (RCB 2/3 level) exhibits immunosuppressive features^[8,20,33]^. Based on these findings, we believe that immune checkpoint inhibitors have potential application value in breast cancer NAT. For patients with immunosuppressive microenvironments, the use of immune checkpoint inhibitors, such as PD-1/PD-L1 inhibitors may help restore immune responses and improve the efficacy of NAT^[7,47]^.

Our study elucidated the associations between specific cellular subtypes in the breast cancer tumor microenvironment and NAT response through multimodal data integration^[52–54]^. The results demonstrated that the abundance of mast cells at the tumor-invasive margin and their subtype expression profiles were critically associated with NAT resistance. The elevated expression of resting mast cells in the NAT-resistant group may contribute to therapeutic resistance by releasing immunosuppressive factors (e.g., IL-1, TGF-β)^[55,56]^, thereby inhibiting antitumor immunity^[53,57]^. Concurrently, the dual prognostic impact of macrophage polarization states aligned with previous findings: the higher proportion of M1-polarized macrophages in the NAT-sensitive group underscored their proactive role in activating cellular immunity and promoting tumor clearance^[44,53,54,57]^. Conversely, the increased M2-polarized macrophages in the NAT-resistant group correlated with immunosuppression, potentially attenuating immunotherapy efficacy through secretion of anti-inflammatory cytokines and facilitation of tumor angiogenesis^[53,58]^. Furthermore, corroborating evidence indicates that immunotherapy-activated T cells can drive macrophage skewing toward terminally differentiated M1-like subtypes^[53,59]^, which are essential for achieving immune activation effects and enhancing therapeutic outcomes – a finding consistent with our results. From a clinical translation perspective, resting mast cells and M2 macrophages may serve as combinatorial biomarkers for predicting NAT efficacy, while modulating their functional states could potentiate therapeutic sensitivity^[56,58]^.

The AI model developed in this study, by integrating multimodal data, provides a comprehensive predictive framework that can effectively guide clinical treatment decisions. The model identifies histopathological features related to treatment response and reveals the mutational status of immune cells through immunological analysis^[30]^. In the detection of tumor heterogeneity, compared with the NAT-resistant group, the NAT-sensitive group had significant increases in the proportion of mutation changes, the rate of silent mutations, the rate of nonsilent mutations, homologous recombination deficiency, and SNV neoantigens, highlighting the prognostic value of mutation immune monitoring in predicting pathological complete response^[43,60]^. Moreover, the detection of HRD status also provides important references for new drug selection^[60]^. For instance, for HRD-positive breast cancer patients^[60]^, the use of poly (ADP-ribose) polymerase (PARP) inhibitors may achieve better therapeutic effects^[61]^, providing theoretical basis for the selection of clinical new drugs and the formulation of individualized treatment plans^[60,62,63]^. Our study further reveals the dynamic correlation between NAT sensitivity and the distribution characteristics of TILs in breast cancer^[24,38]^. Specifically, compared with the NAT-resistant group, the expression of TILs in the NAT-sensitive group (achieving pathological complete response) was significantly higher, indicating that elevated TILs expression is associated with good prognosis in breast cancer patients^[31,36,38,51]^. By further exploring the spatial distribution patterns of tumor-infiltrating lymphocytes, we can better understand the effects of NAT on the tumor immune microenvironment and provide new therapeutic strategies for understanding treatment resistance mechanisms^[45]^. Relevant studies have shown that the quantitative level of TILs, spatial organization, and the formation of lymphoid aggregates all show potential as prognostic and predictive biomarkers for chemotherapy and immunotherapy responses^[11,64]^. Although this study has made breakthroughs in preoperative prediction, it still faces challenges such as prediction bias caused by the heterogeneity of biopsy samples and the limited number of training sets affecting the model’s generalization ability. External validation was implemented, yet the two center design limits racial diversity, molecular-subtype coverage, and neoadjuvant regimen heterogeneity – potentially undermining generalizability. Prospective multicenter validation is therefore warranted to verify performance in diverse populations^[65]^. While scanner-specific bias was mitigated by unified RGB-channel standardization, residual batch effects arising from inter-run staining variability remain plausible^[35]^. Future multicenter, multiscanner studies that incorporate daily quality control slides and generative-adversarial-network-based (GAN) stain normalization are warranted to minimize batch variability and satisfy the standardization prerequisites for clinical deployment^[]^. Though the model demonstrated promising risk-stratification capacity in the retrospective cohort, its application to surgical de-escalation decisions must be approached cautiously^[66]^. Multicenter prospective studies are required to validate robustness across diverse populations and to integrate model outputs with established clinicopathological variables (e.g., TNM stage and tumor differentiation grade) to develop a comprehensive decision-support framework^[67,68]^. Despite its favorable predictive performance, the model faces substantial technical barriers to clinical implementation, including high computational demands, limited interoperability among digital-pathology platforms, and complex workflow integration^[69]^. To facilitate smooth integration into routine pathology workflows, we will pilot a lightweight cloud-based solution accompanied by standardized quality control protocols and targeted training, thereby enabling the stepwise deployment of an interpretable AI model.

## Conclusion

This study developed an interpretable AI model that uses H&E slides to predict response to NAT in breast cancer. The model’s predictions are biologically interpretable, correlating with tumor microenvironment dynamics and spatial TIL patterns, which offers a novel strategy for personalizing NAT.

## Data Availability

The authors declare that they had full access to all of the data in this study and take complete responsibility for the integrity of the data and the accuracy of the data analysis. The datasets generated or analyzed during the current study involve potential patient privacy. Therefore, they are not publicly available but can be requested from the corresponding author upon reasonable request.

## Research registration unique identifying number (UIN)

Name of the registry: Prediction of Neoadjuvant Therapy Response in Breast Cancer Based on Interpretable Artificial Intelligence. Unique identifying number or registration ID: ChiCTR2500105600 (PID: 274865). Hyperlink to your specific registration (must be publicly accessible and will be checked): https://www.chictr.org.cn/searchproj.html?page=3&title=&officialname=&subjectid=&regstatus=&regno=&secondaryid=&applier=&studyleader=&createyear=&sponsor=&secsponsor=&sourceofspends=&studyailment=&studyailmentcode=&studytype=&studystage=&studydesign=&recru
